# Neuroprotective Effects of Doxycycline in the R6/2 Mouse Model of Huntington’s Disease

**DOI:** 10.1007/s12035-019-01847-8

**Published:** 2019-12-26

**Authors:** Emanuela Paldino, Claudia Balducci, Pietro La Vitola, Luisa Artioli, Vincenza D’Angelo, Carmela Giampà, Vladimiro Artuso, Gianluigi Forloni, Francesca R. Fusco

**Affiliations:** 1 IRCCS Fondazione Santa Lucia, Laboratory of Neuroanatomy, Via del Fosso di Fiorano, 64, Rome, Italy; 2 Department of Neuroscience, Istituto di Ricerche Farmacologiche Mario Negri IRCCS, Milan, Italy; 3 Department of Neuroscience, University of Rome Tor Vergata, Rome, Italy; 4 ULSS 3 Serenissima, Via Don Tosatto, 147-30174 Venice, Italy

**Keywords:** Huntington’s disease, Doxycycline, Inflammation, Neurodegeneration, pCREB, BDNF, Microglia

## Abstract

Mechanisms of tissue damage in Huntington’s disease involve excitotoxicity, mitochondrial damage, and inflammation, including microglia activation. Immunomodulatory and anti-protein aggregation properties of tetracyclines were demonstrated in several disease models. In the present study, the neuroprotective and anti-inflammatory effects of the tetracycline doxycycline were investigated in the mouse model of HD disease R6/2. Transgenic mice were daily treated with doxycycline 20 mg/kg, starting from 4 weeks of age. After sacrifice, histological and immunohistochemical studies were performed. We found that doxycycline-treated R6/2 mice survived longer and displayed less severe signs of neurological dysfunction than the saline-treated ones. Primary outcome measures such as striatal atrophy, neuronal intranuclear inclusions, and the negative modulation of microglial reaction revealed a neuroprotective effect of the compound. Doxycycline provided a significantly increase of activated CREB and BDNF in the striatal neurons, along with a down modulation of neuroinflammation, which, combined, might explain the beneficial effects observed in this model. Our findings show that doxycycline treatment could be considered as a valid therapeutic approach for HD.

**Electronic supplementary material:**

The online version of this article (10.1007/s12035-019-01847-8) contains supplementary material, which is available to authorized users.

## Introduction

Neurodegeneration of striatal projection neurons is the main event in the pathology of Huntington’s disease (HD). Although the gene was discovered in 1993, many mechanisms determining neuronal death have been involved over the years. In the last decade, the role of neuroinflammation has gained momentum.

Inflammation is a physiological response aimed at repairing damaged tissue in several different conditions. Indeed, inflammation is designed to initiate healing processes, and thus protect the organism [1, 2]. A major role in central nervous system is played by microglia. Activated microglia is able, in fact, to produce large quantities of dangerous compounds such as prostaglandin E2 (PGE2), nitric oxide (NO), and cytokines like tumor necrosis factor-a (TNF-a), interleukin-1 b (IL-1 b), and interleukin 6 (IL-6) that participate in the pathological processes through the activation of nuclear factor-K B (NF-k B), leading to neurodegeneration [3, 4].

Thus, the inhibition of aberrant microglial activation through a reduction of pro-inflammatory factors could be desirable, in the effort of halting neuronal degeneration, in diseases such as HD and other neurodegenerative disorders. One of the emerging pharmacological approaches aimed at reducing neuroinflammation involves the use of tetracyclines [5, 6].

The interest for tetracyclines in neurodegenerative diseases originated from the discovery of their anti-amyloidogenic activities about 20 years ago [7, 8]. It was, indeed, widely demonstrated that tetracyclines can inhibit the aggregation of both prion protein (PrP) and β-amyloid peptide. In addition, they enhance their sensitivity to protease digestion, thus favoring their degradation. In vitro experiments show that tetracyclines prevent PrP 106-126-mediated neurotoxicity and astroglial proliferation [8]. In vivo studies, on the other hand, demonstrated that tetracyclines reduced infectivity, delayed the onset of pathology, and prolonged survival of Syrian hamster infected with the pathological form of PrP [9–11].

The immunomodulatory properties of tetracyclines were clearly demonstrated in various conditions (reviewed in [12]). In this study, we will focus our attention on doxycycline, a second generation tetracycline endowed with both anti-amyloidogenic and anti-inflammatory effects, with a more favorable BBB penetration and a safer toxicological profile. Immunomodulatory properties of tetracyclines were demonstrated in diseases such as experimental autoimmune encephalomyelitis (EAE) and focal ischemia [13–15].

More recently, Balducci and coworkers [16, 17] demonstrated that doxycycline could counteract the deleterious actions of β-amyloid oligomers (AβOs), recognized as the main detrimental species of Alzheimer’s disease (AD), on memory. Such effect was associated with an anti-inflammatory action in both an AβO-induced acute mouse model, and in the APP/PS1dE9 chronic mouse model of AD.

Doxycycline has also been investigated in neurodegenerative diseases involving clinical studies. Indeed, it was observed that early Creutzfeldt-Jakob disease patients treated with doxycycline survived longer [18–20]. Moreover, our group is currently performing a 10-year preventive clinical trial in subjects at genetic risk of developing the prion disease fatal familial insomnia. Furthermore, positive outcomes were also observed in multiple sclerosis patients treated with a combination of interferon-β and doxycycline [21].

Thus, based on all this encouraging evidence, we aimed at investigating the possible action of doxycycline in the R6/2 mouse model of HD. The compound had already been tested in a previous study [22], yielding, however, to rather disappointing results. Here, we evaluate the efficacy of doxycycline on multiple core therapeutic targets in the effort of better understanding the therapeutic potential of doxycycline in HD as well as the possible mechanisms of neuroprotection involved.

## Materials and Methods

### Animals

All animal experiments, which satisfy ARRIVE guidelines, were performed in accordance with European Communities Council Directive (2010/63 EU) as adopted by the Santa Lucia Foundation Animal Care and Use and approved by Italian Ministry of Health. Transgenic female R6/2 mice carrying the mutant human HTT exon 1 which determine the abnormal expanded CAG repeats were kept in coupling with B6CBAF1/J males, all obtained from Jackson Laboratories (Bar Harbor, ME). Animals were pathogens free, including common pathogens such as Helicobacter. F1 mice were used to perform all experiments. Genotyping occurred at 21 days of age, mice were weaned and the treatments started.

### Treatment Schedule

Wild type and R6/2 mice (13 mice/per experimental group) were treated intraperitoneally with either vehicle (0.9% saline) or doxycycline dissolved in saline (20 mg/kg/day). Doxycycline was administered twice a day until sacrifice, at the concentration of 10 mg/kg in order to ensure the persistent presence of the antibiotic. Animals were identified by a randomly assigned code and housed 4 per cage under conventional laboratory conditions (room temperature 20 ± 2 °C; humidity 60%) and a 12/12 h light/dark cycle (7:00 am–7:00 pm) with ad libitum access to food and water. All the experimental data were collected by observers who were blinded to genotype and treatment.

### Survival and Weight

The survival study, according to Hersch and Ferrante [23], was conducted following the criterion for euthanasia which is the point when animals were not able to right themselves after 30 s when placed on their side. All experimental mice were weighed twice a week starting from the beginning of treatment until sacrifice. Their weight was recorded and weight variations were calculated and plotted. Day 28 represents the first day of the 4th week from the beginning of treatment.

### Behavior

#### Clasping

R6/2 mice exhibit a hind-limb clasping phenotype when suspended by the tail. The clasping phenotype has been extensively studied and used to recognize the neurological impairment in HD mice, and it is considered a measure of disease progression. Mice were suspended by their tail for 60 s. The total time spent clasping the hind-limb was recorded twice weekly.

#### Rotarod

The five-station rotarod performance test (Rotarod/RS LSI Letica, Biological Instruments, Varese, Italy) was used to estimate mouse motor coordination and balance. Tests were performed by placing mice on a horizontally rotating rod, which is low enough to prevent animal damages, but high enough to induce the fall. Mice performed rotarod test twice weekly from 4th to 13th weeks of age. Three-trial measurements on the rod for their latency or fall were recorded. A maximum latency of 60 s was defined for mice that did not fall.

#### Open Field

Motor activity and anxiety were measured in an open field consisting in a circular arena with a 60 cm diameter and a white floor divided into central and peripheral areas by drawing black line. The open field measurements were performed in a soundproof room illuminated by an 80-W red ceiling light. The video camera on the arena was connected to a video recorder of a computer placed in the next room. Mice were placed into the arena for 10 min, while distance traveled and velocity were recorded and analyzed by a specific software (Noldus, Wageningen, the Netherlands).

### Primary Neurodegeneration Outcomes

#### Analysis of Gross Striatal Area and Volume

Standard Nissl staining was performed on coronal step serial sections from rostral neostriatum through the level of anterior commissure (interaural 4.66 mm/bregma 0.86 mm to interaural 3.34 mm/bregma − 0.46 mm) from 6 animals per group. Gross striatal volume was measured using Neurolucida Stereo Investigator software (Zeiss, Cochester, VT, USA) running the Cavalieri estimator probe.

### Evaluation of NIIs Number and Size

All brain sections were processed for single label EM-48 ubiquitin (Chemicon, Temecula, CA) immunofluorescence and counterstained with Neurotrace™ to calculate the number of neurons containing intranuclear inclusions (NIIs). The nucleus of each neuron was examined to ascertain the presence of NIIs. All neurons in each hemisphere for each brain section of all mice (*n* = 13/treatment group) were analyzed to determine the number, intensity of immunofluorescence and size of NIIs in the striatal neurons in both saline and doxycycline-treated R6/2 mice (wild-type littermates did not show NIIs–like ubiquitin immunoreactivity, data not shown). Images were acquired with a 40× and 63× objective on a confocal laser scanner microscopy (Zeiss LSM 800) under no saturating exposure conditions. The same acquisition setting was performed for each sample.

### Microglial Morphology

Microglial morphology was studied by immunostaining brain sections with an antibody labeling microglia (1:500 goat anti-Iba-1 from Bionovus). Striatal brain sections were incubated with the primary antibody for 72 h at 4 °C, followed by incubation with the secondary antibody for 2 h at room temperature. Images were acquired with confocal laser scanner microscopy (Zeiss LSM 800) in order to perform soma size analysis. Microglia cells in the area of interest were captured using a 40× objective producing images in the format 1024 × 1024, and Airy Units 1.0. This configuration was used for all samples. Collected images were exported in TIFF format, brightness and contrast were adjusted. The protective or toxic phenotype was characterized by using 63× Z-stack images, performing the school analysis available in the Java image processing and analysis program ImageJ version 8. The Iba-1 immunostained area was calculated ad Iba-1 area/total area analyzed and indicated as percentage.

### Immunofluorescence Analysis

Brain tissue sections were incubated with primary antibodies for 72 h at 4 °C, followed by incubation with secondary antibodies for 2 h at room temperature. Neurons were counterstained for their visualization with Neurotrace™. The primary antibodies used were anti-pCREB (1:200 polyclonal pCREB, Millipore, Italy), BDNF (1:200 polyclonal anti-BDNF, Novus Biologicals, Italy), GFAP (1:600 polyclonal anti-GFAP, Millipore, Italy), and PSD95 (1:100 mouse anti-PSD95, Abcam, Italy). The secondary antibodies used were Alexa Fluor 488 and Alexa Fluor 555 (Jackson). Brain sections all at the same bregma level were mounted on gelatin-coated slices, cover slipped with GEL-MOUNT. Samples were examined with the support of confocal laser scanner microscopy (Zeiss LSM 800), images were acquired and subsequently analyzed to quantify the immunofluorescence intensity of phosphorylated CREB and BDNF, as well as the number of PSD95- and GFAP-positive cells. Phosphorylated CREB and activated BDNF immunofluorescence quantification was carried out by using the Java image processing and analysis program, ImageJ version 8. We selected cells of interest using a circle selection tool. From the Analyze Menu Set measurement, we selected “Mean Grey Value,” “Area,” and “Min&Max Grey Value.” The region next to cells with no fluorescence was considered “background” and subtracted. Finally, the “Measure” tool was selected from the Analyze menu and a mean value was obtained.

GFAP and PSD95 positive cell count was performed using images acquired at the confocal laser scanner microscope (Zeiss LSM 800). For each section, the striatum was subdivided in five representative fields using × 40 and × 63 magnification for GFAP and PSD95 under non saturating exposure conditions and using the same acquisition settings for all samples. Gain and laser power were selected at specific value to allow optimal visualization of the fluorophore used as secondary antibody and standardized using sections from wild-type mice. These settings were applied as standard for subsequent images. Using a 63× objective, Z-stacks images of GFAP were collected using computer controlled microstepper stage of the confocal microscope to quantify the area of GFAP positive cells. The GFAP immunostained area was calculated as GFAP immunostained area/the total area analyzed and indicated as staining percentage area.

## Statistical Analysis

The data collected were analyzed to compare the effect of doxycycline on weight, clasping, rotarod, open field as well as NIIs percentage, pCREB, BDNF, GFAP, PSD95, and Iba-1 expression in the striatum of differently treated mouse groups. Statistical analysis was performed by ANOVA available on the software GraphPad Prism version 8.0. *p* values < 0.05 were considered statistically significant. Survival data were analyzed by means of a product limit method of Kaplan and Meier and *p* value was set at 0.001 for significant results.

## Results

### Doxycycline Increases R6/2 Mouse Survival and Reduces Weight Loss

**Fig. 1:**
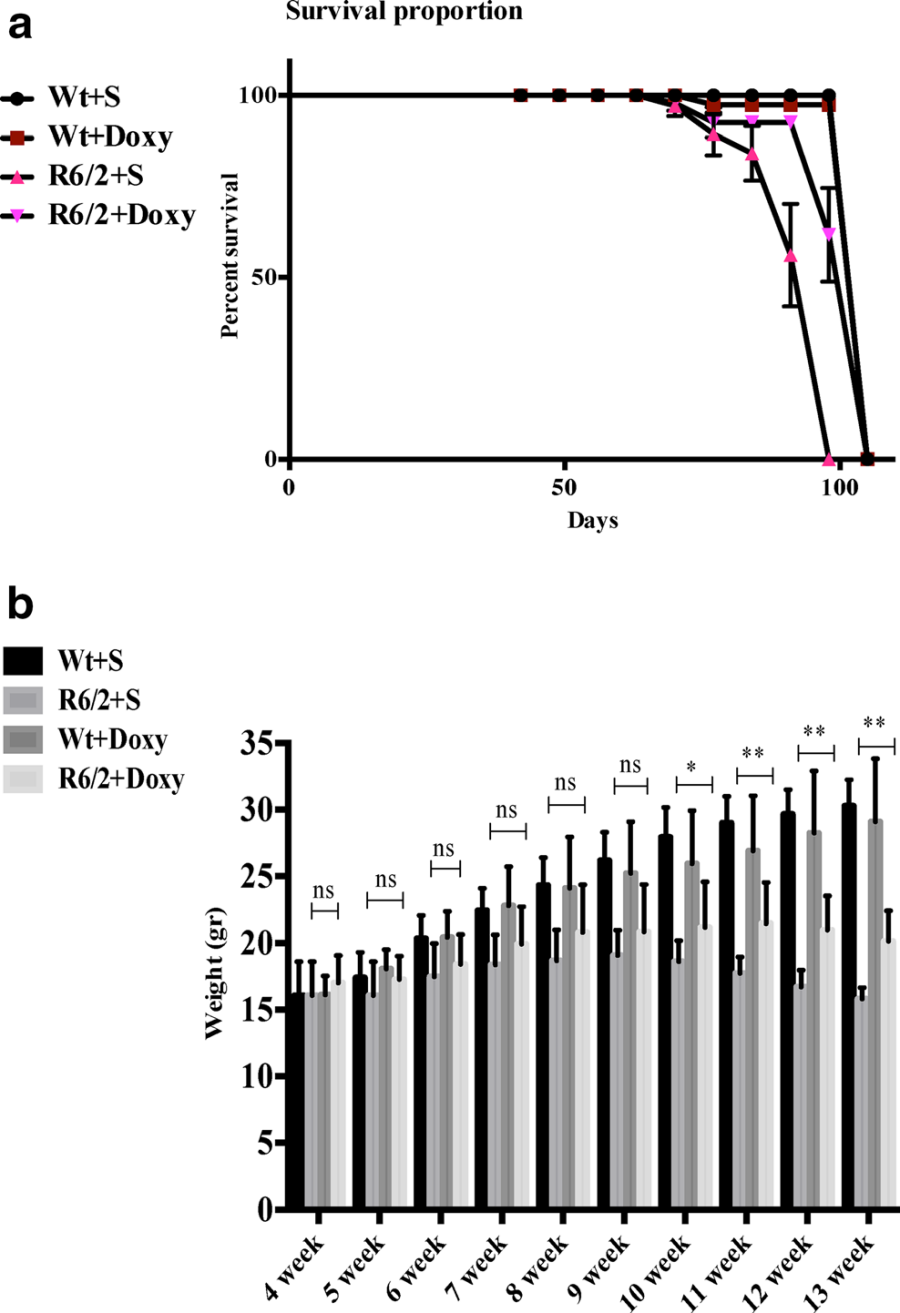
Survival and weight in R6/2 mice treated with doxycycline or saline. **a** Kaplan-Meier curve of survival. R6/2 mice treated with doxycycline showed a mean survival time that was significantly (*p* < 0.001) longer than that of R6/2 mice treated with saline. **b** A three-way ANOVA shows a significant effect of genotype (*F*_1,140_ = 579.5; *p* < 0.0001), treatment (*F*_1,140_ = 15.13; *p* < 0.01), time (*F*_9,140_ = 61.23; *p* < 0.01), and a genotype × treatment × time interaction (*F*_1,140_ = 58.15; *p* < 0.001) in the weight of R6/2 mice treated with doxycycline

Doxycycline treatment promoted a longer survival of R6/2 mice, as shown by the Kaplan-Meier curve. In our study, R6/2 mice were followed weekly until death. Saline-treated R6/2 mice, expressed as a percentage of survival, died between days 84 and 91, whereas wild-type mice and doxycycline-treated R6/2 survived 2 weeks longer as shown in Fig. 1a.

The effect of treatments and genotype on mouse weight is illustrated in Fig. 1b. The differences among groups were not significant until 10 weeks of age. At 11 weeks, wild-type mice treated with saline weighed 32 ± 0.50 g and were not significantly different from wild type treated with doxycycline. In the fully symptomatic stage, R6/2 mice receiving saline showed a major weight loss (weighing 17 ± 0.85 g), while R6/2 treated with doxycycline weighed 21 ± 0.35 g, indicating a significant effect of the drug.

### Doxycycline Improves Neurological Deficits in R6/2 Mice

#### Clasping

**Fig. 2:**
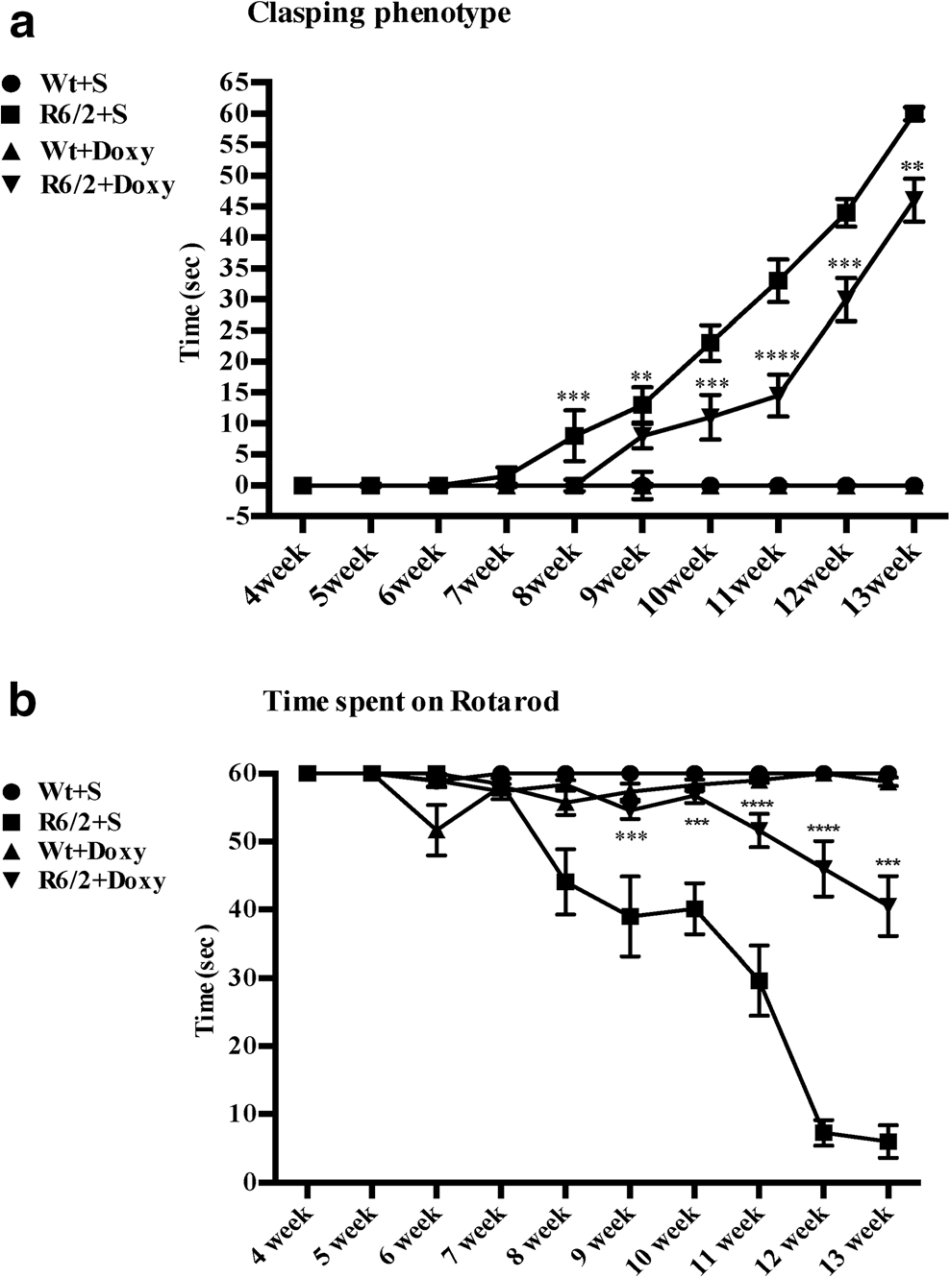
Hind-limb clasping phenotype, rotarod in R6/2 mice treated with doxycycline. **a** Data analysis indicates a statistically significant effect of treatment and time. Doxycycline treatment in the R6/2 mice significantly reduced clasping phenotype during animal’s aging *F*_1,560_ = 742.2 *p* < 0.001. Genotype effect *F*_1,560_ = 42.52 *p* < 0.0001, time × genotype *F*_9,560_ = 4.420 *p* < 0.001, time × treatment *F*_9,560_ = 122.2 *p* < 0.001, genotype × treatment *F*_1,560_ = 42.52 *p* < 0.001. **b** A three-way ANOVA with genotype, treatment and time as main factors, revealed that R6/2 mice have a statistically significant impairment in motor coordination respect to wild-type mice *F*_1,560_ = 296.6 *p* < 0.00001 and that doxycycline treatment improves performance *F*_1,560_ = 81.25; *p* < 0.0001

Paw clasping occurred only after 8 weeks of age in R6/2 mice. Figure 2a shows that the time spent clasping was significantly less in the doxycycline-treated mice than in the saline group, genotype × treatment *F*_1,560_ = 47.60 *p* < 0.001. Clasping is absent in saline or doxycycline-treated wild-type mice as shown in the graph. In saline-treated R6/2 mice, the clasping phenotype was significantly evident at 8 weeks of age and then developed progressively reaching the maximal levels by 13 weeks of age, when the mice are fully symptomatic. Doxycycline-treated R6/2 mice developed later the clasping response, which was significantly reduced in the later stages of the disease, with respect to saline-treated R6/2 mice (time × treatment *F*_9,560_ = 120.4 *p* < 0.001).

#### Motor Behavior

Motor behavior performances of mice were evaluated by rotarod apparatus. R6/2 mice had a statistically significant impairment in motor coordination compared to wild-type mice *F*_1,560_ = 296.6 *p* < 0.0001; the three-way ANOVA showed a significant improvement of motor performance *F*_1,560_ = 81.25 *p* < 0.0001 (Fig. 2b) after doxycycline treatment in R6/2 mice.

**Fig. 3:**
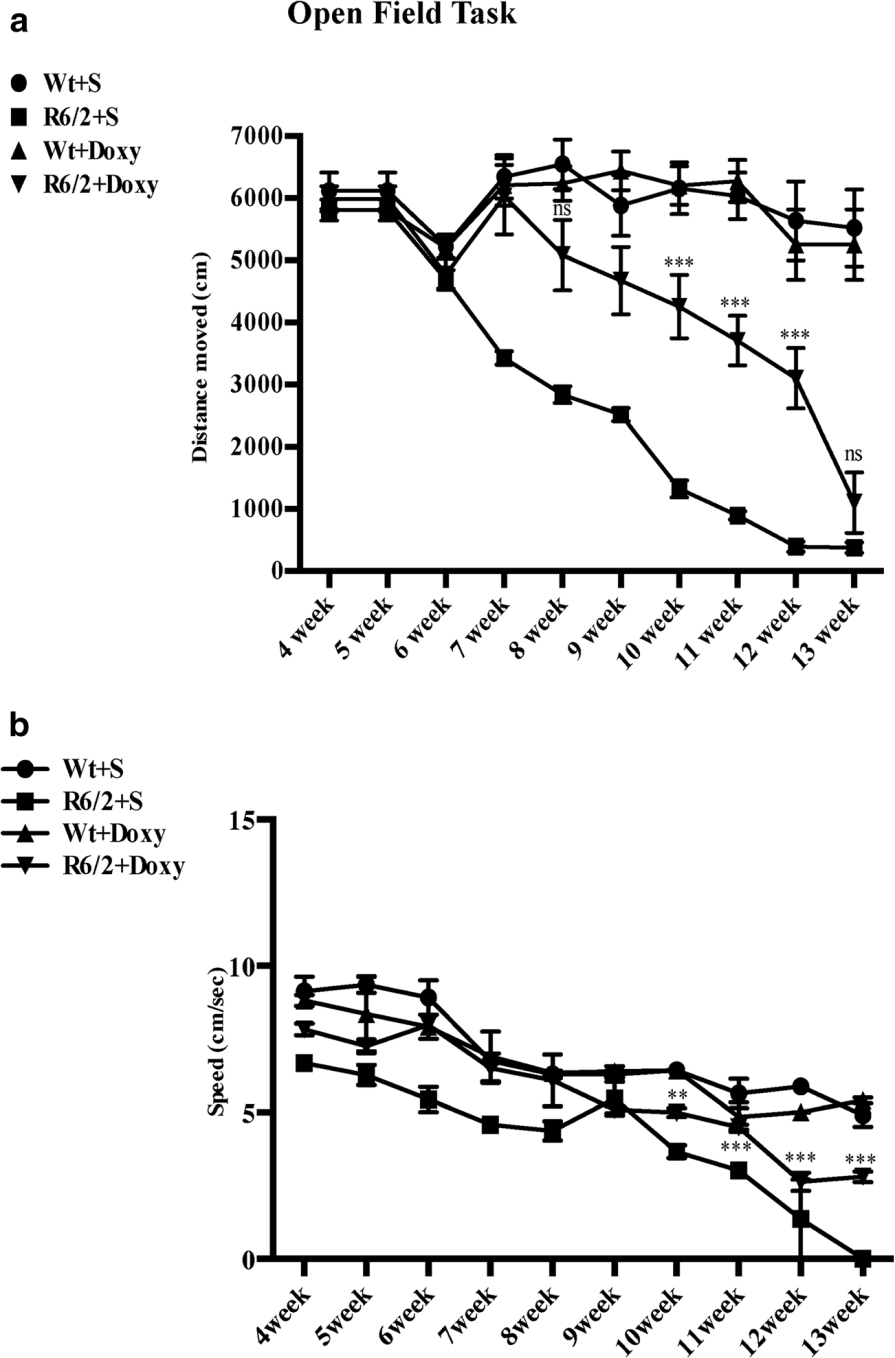
Open field task in R6/2 mice treated with doxycycline. Graphs show motor activity data collected in the open field task including the total distance traveled and speed of locomotion in the arena. **a** R6/2 mice traveled a shorter distance compared to wild-type mice (genotype effect with *F*_1,560_ = 54.79; *p* < 0.0001), which was recovered in doxycycline-treated R6/2 mice. **b** Histograms show mouse speed in the open field. As observable R6/2 mice display a significant lower speed compared to wild-type mice. Doxycycline treatment rescued R6/2 speed (significant genotype × treatment interaction *F*_1,11_ = 79.78; *p* < 0.001) until 12 weeks of age

Motor activity was further investigated in the open field task (Fig. 3a, b), including the total distance traveled and speed of locomotion in the arena. R6/2 mice traveled a shorter distance at a lower speed than wild-type mice (genotype effect *F*_1,560_*p* < 0.001).

Conversely, doxycycline treatment was able to promote the rescue of motor performances in a genotype-dependent fashion (significant genotype × treatment interaction *F*_1,560_ = 144.1 *p* < 0.001), traveling a longer distance respect to saline-treated R6/2 (treatment × genotype *F*_1,560_ = 58.71 *p* < 0.001).

#### Doxycycline Reduces the Extent of Neuropathology in R6/2 Mice

##### Striatal Area

**Fig. 4:**
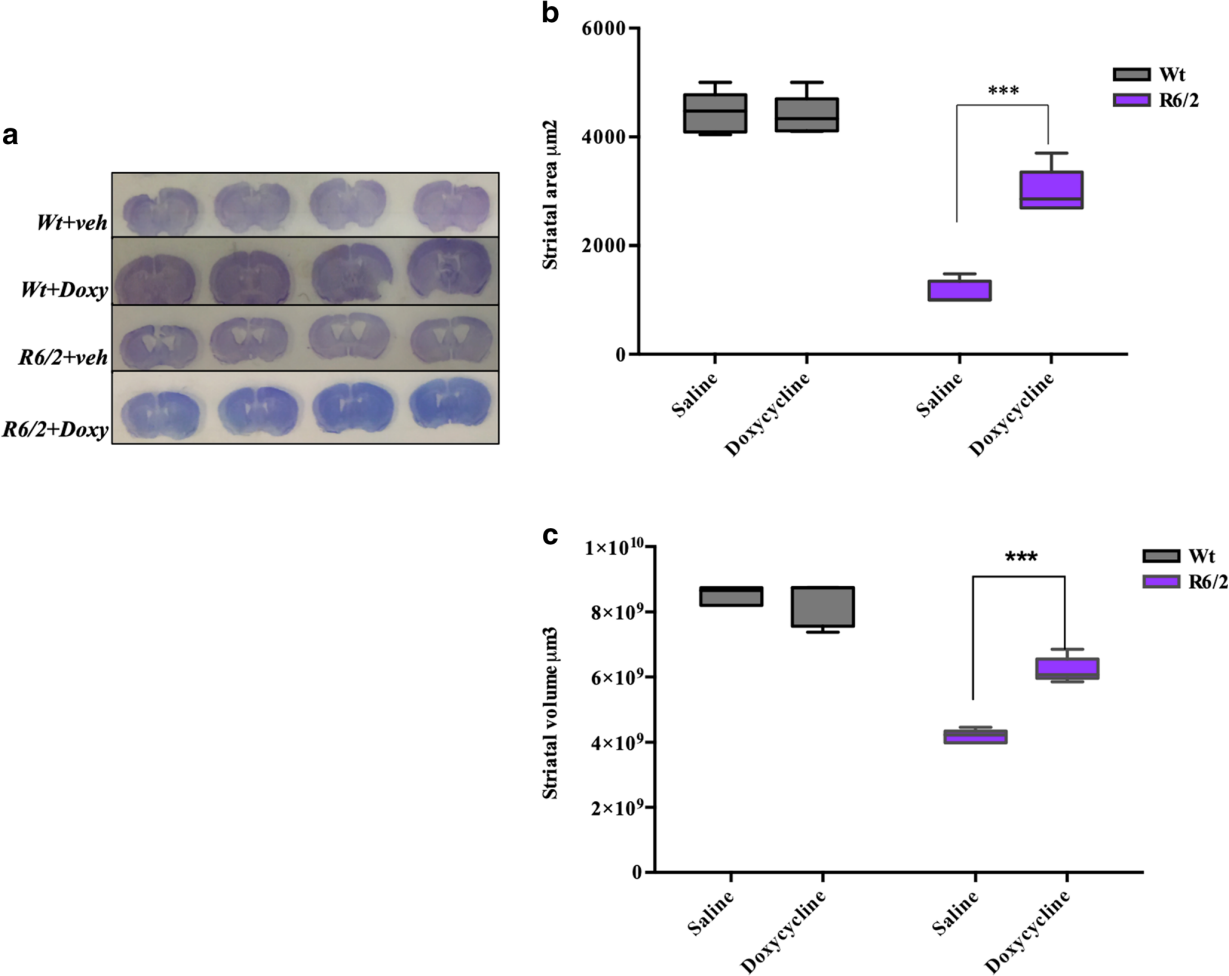
Doxycycline treatment reduced striatal atrophy in R6/2 mice. **a** Transmitted light microscope images showing Nissl-stained coronal sections of representative saline- or doxycycline-treated wild-type, and saline or doxycycline-treated R6/2 mice. R6/2 mice treated with saline display marked gross striatal atrophy and enlarged ventricles compared to R6/2 mice treated with doxycycline. **b** Histograms are mean ± SEM of striatal area quantification. Two-way ANOVA showed a statistically significant effect of genotype (*F*_1,16_ = 223.0; *p* < 0.0001), treatment (*F*_1,16_ = 32.63; *p* < 0.001) and genotype × treatment interaction *F*_1,16_ = 36.54; *p* < 0.001). **c** Histograms are mean ± SEM of striatal volume quantification. R6/2 mice treated with saline had a statistically significant reduced striatal volume compared to wild type. Striatal volume of R6/2 mice treated with doxycycline was significantly greater than that of saline-treated R6/2 mice *F*_1,16_ = 23.45 *p* = 0.0002

Stereology analyses showed that the striatal area of saline-treated R6/2 mice was smaller than wild-type animals (with an average area of 1000 μm^2^ compared to 5454 μm^2^ of the wild-type littermates). Treatment of R6/2 mice with doxycycline prevented striatal area reduction (4026.98± 0.38 × 10^5^ μm^2^) as shown in Fig. 4b (*p* < 0.017).

##### Striatal Volume

The gross striatal volume of vehicle treated R6/2 mice was strongly reduced compared to their wild-type littermates (Fig. 4c). The mean striatal volume was 5.18± 0.27 × 10^7^ μm^3^ in the saline-treated R6/2 mice, whereas it was 10.01± 0.20 × 10^7^ μm^3^ in the wild-type animals. The striatal volume of doxycycline-treated R6/2 mice was 7.98± 0.20 × 10^7^ μm^3^ (*p* < 0.0022).

##### Doxycycline Reduced NIIs in the R6/2 Striatal Neurons

**Fig. 5:**
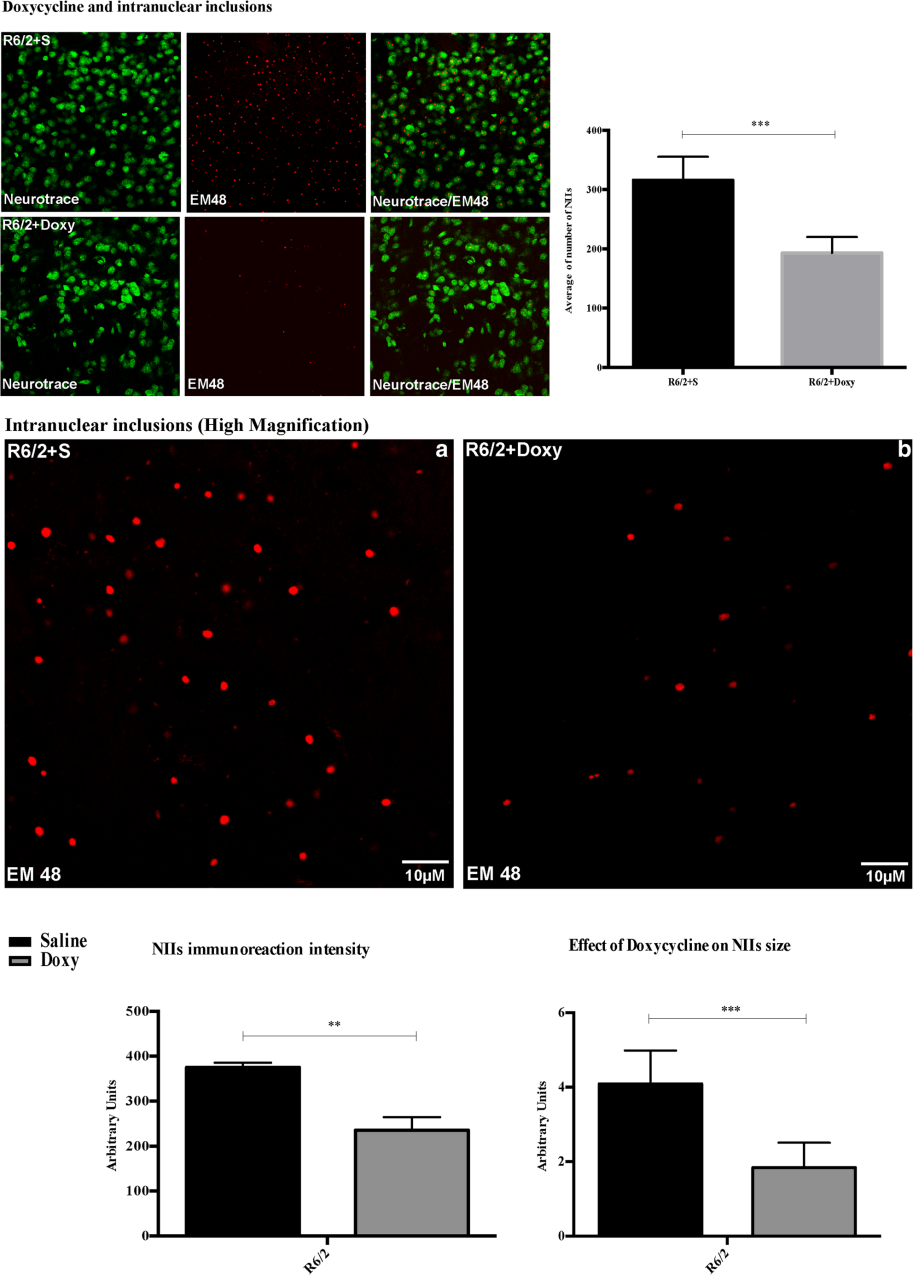
Doxycycline reduces NIIs number, immunostaining intensity and size in R6/2 mice. Two-way ANOVA analysis performed on data obtained by saline- and doxycycline-treated R6/2 mice (*n* = 8 female and *n* = 5 male mice for each group; 4 brain sections for mice) revealed a statistically significant effect of treatment on NIIs number, intensity and size. *Bonferroni* analysis showed a significant decrease of NIIs number (*p* < 0.001), intensity (*F*_1,48_ = 59.67; *p* < 0.01) and size in mice treated with doxycycline respect to saline-treated R6/2 mice (*F*_1,48_ = 60.82; *p* < 0.001)

The expression of exon1 of mutant huntingtin in the R6/2 mice results in the formation of neuronal intranuclear inclusions (NIIs) detected with the antibody EM-48. The analysis of EM-48 immunofluorescence in the striatal brain region of 13-week-old R6/2 mice treated with doxycycline, counterstained with Neurotrace™, showed that the number of NIIs was significantly reduced compared to the saline-treated group (Fig. 5). Moreover, the analysis of all doxycycline-treated R6/2 mice revealed that the intensity of NIIs immunoreaction and its area were decreased compared to that of saline-treated mice (Fig. 5a, b high magnification). This strong reduction in the number and size of EM-48 immunoreactive NIIs confirmed that daily administration of doxycycline reduced the development of neuronal inclusions in R6/2 mice.

##### Doxycycline Prevented the Decrease in CREB Activation in Striatal Neurons of R6/2 Mice

**Fig. 6:**
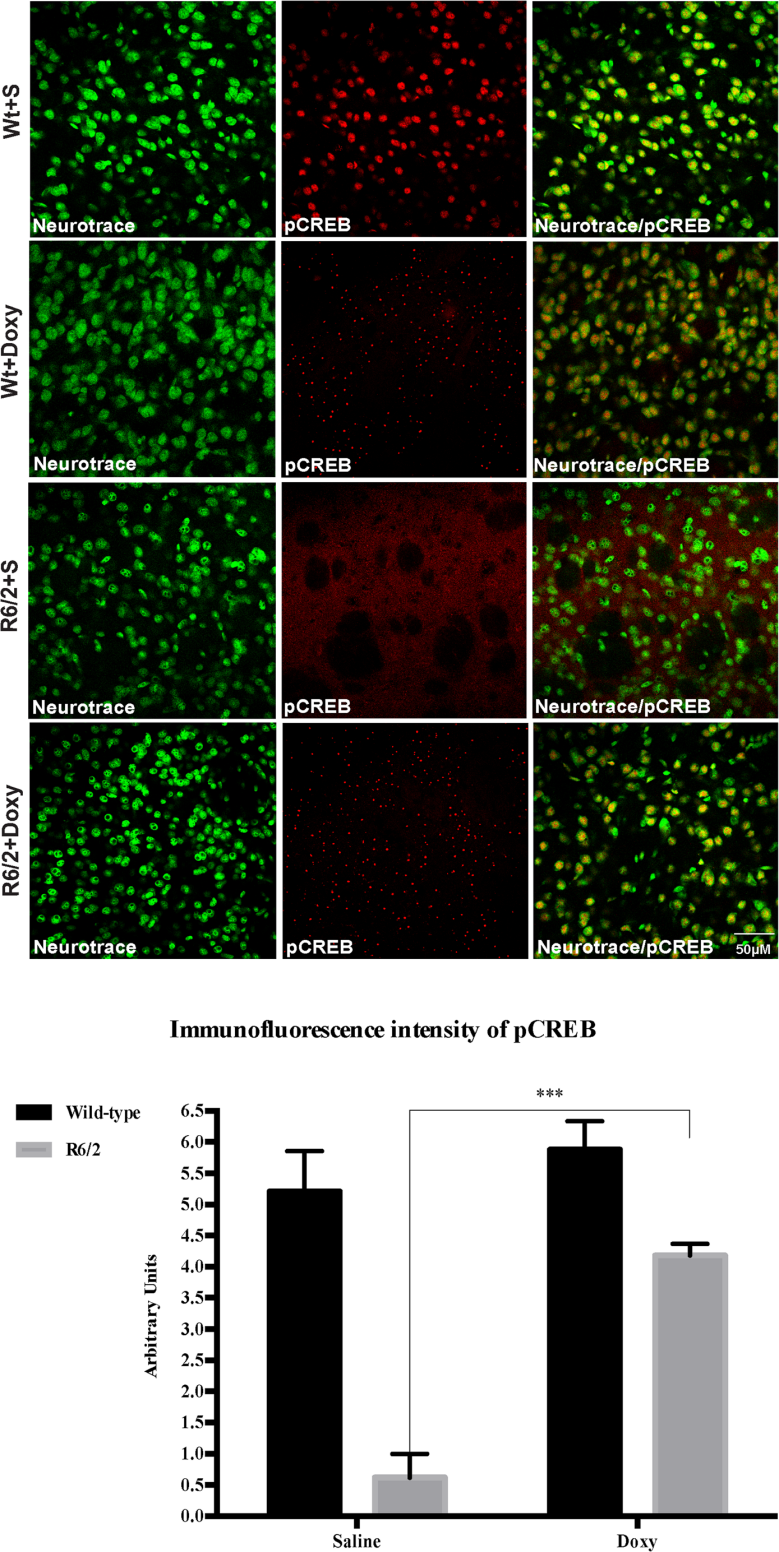
Doxycycline induces neuroprotection in R6/2 mice. Images are confocal laser scanning microscopy acquisitions of double-label immunofluorescence for pCREB (visualized in red-Cy3 fluorescence) and the Nissl-like fluorescent marker Neurotrace (visualized in green fluorescence). Collected images show the effect of doxycycline administration in R6/2 mice striatal neurons (*n* = 8 female and *n* = 5 male mice for each group; 5 brain sections for mice). Histograms show pCREB quantification thus the significant effect of doxycycline (*p* < 0.001) in promoting neuronal protection. Doxycycline treatment had no effect in wild-type mice. (*F*_1,48_ = 22.36; *p* < 0.001)

Indeed, we observed that doxycycline-treated R6/2 mice displayed a significantly higher expression of phosphorylated CREB (pCREB) in the surviving spiny neurons of R6/2 mice. The intensity of pCREB, expressed in arbitrary units, was, indeed, significantly lower in the saline-treated R6/2 mice compared to wild-type littermates with a genotype effect *F*_1,48_ = 49.56 *p* < 0.0001, [24–26]. In contrast, pCREB immunoreactivity was significantly more intense in doxycycline-treated R6/2 compared to the saline-treated R6/2 mice with a significant treatment effect *F*_1,48_ = 22.36 *p* < 0.0001 and a significant genotype × treatment interaction *F*_1,48_ = 10.48 *p* < 0.001 as shown in Fig. 6.

##### Doxycycline Prevented BDNF Expression Reduction in R6/2 Mice

**Fig. 7:**
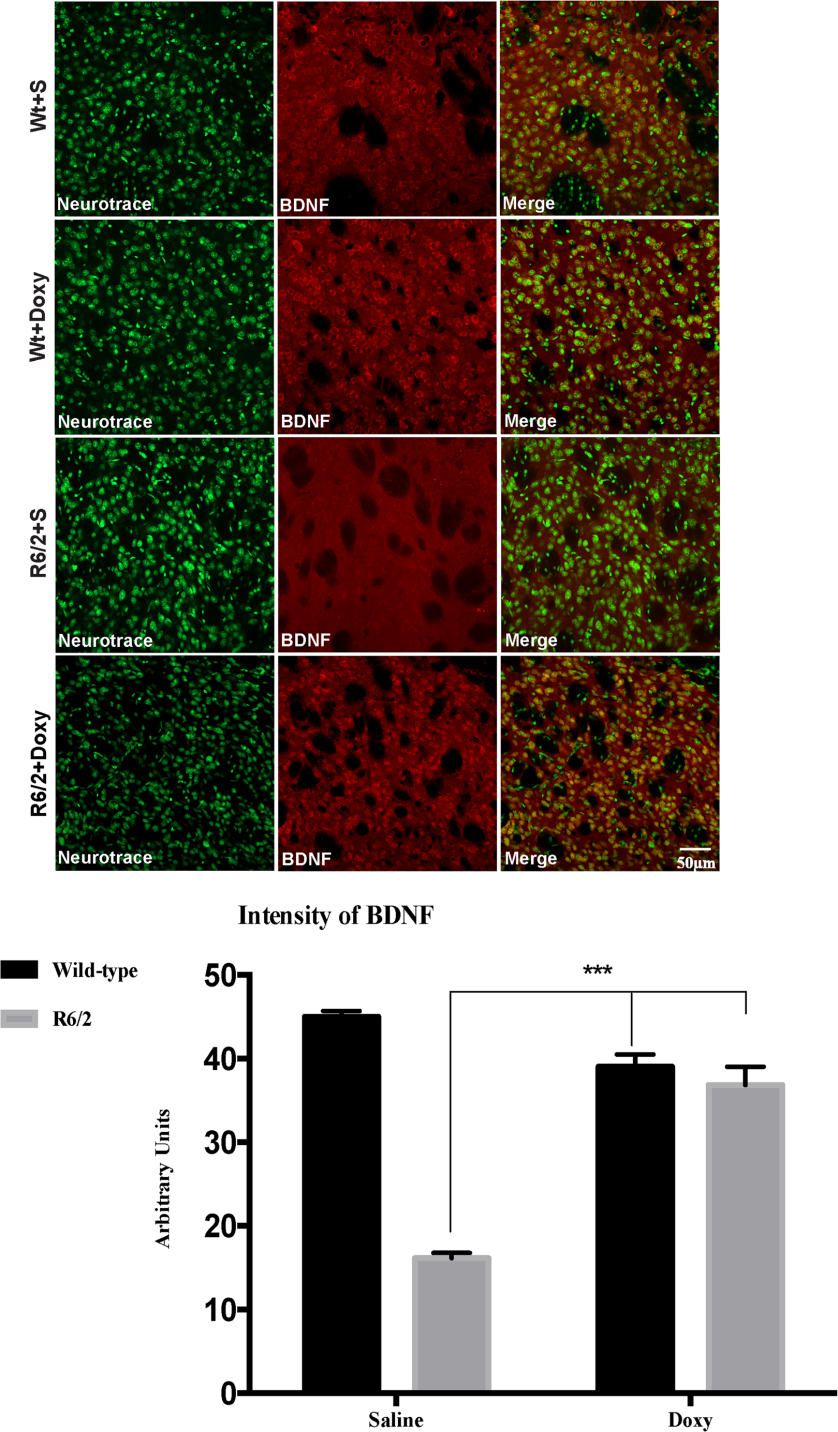
Doxycycline significantly prevents BDNF decrease in R6/2 mice. Images are confocal acquisitions of double-label immunofluorescence for BDNF and Neurotrace. BDNF is showed in red CY-3 fluorescence, Neurotrace is labeled in green. Images show the immunoreaction intensity relative to BDNF in each experimental group. BDNF intensity is drastically decreased in the striatal neurons of R6/2 mice treated with saline *F*_1,48_ = 27.57 *p* < 0.001, but significantly higher, and comparable to wild-type mice, in R6/2 mice treated with doxycycline *F*_1,48_ = 18.96; *p* < 0.001 (*n* = 8 female and *n* = 5 male mice for each group; 5 brain sections for mice). Histograms are mean ± SEM of BDNF quantification

We investigated the effect of doxycycline treatment on BDNF protein expression in the striatum of R6/2 mice. BDNF is a CREB target gene, therefore we aimed at verifying if the above described increase of pCREB was associated with an upregulation of BDNF. As shown in Fig. 7, doxycycline-treated R6/2 showed a significantly higher BDNF protein expression as compared to R6/2 mice receiving saline (Fig. 7) Doxycycline was, therefore, effective in preventing the well described loss of BDNF in HD [27]. Two-way ANOVA indicated a significant effect of genotype *F*_1,48_ = 18.87; *p* < 0.001, a significant treatment effect *F*_1,48_ = 53.63; *p* < 0.001 and a significant genotype × treatment interaction *F*_1,48_ = 56.31; *p* < 0.001.

##### Doxycycline Prevented PSD95 Protein Reduction in R6/2 Mice

**Fig. 8:**
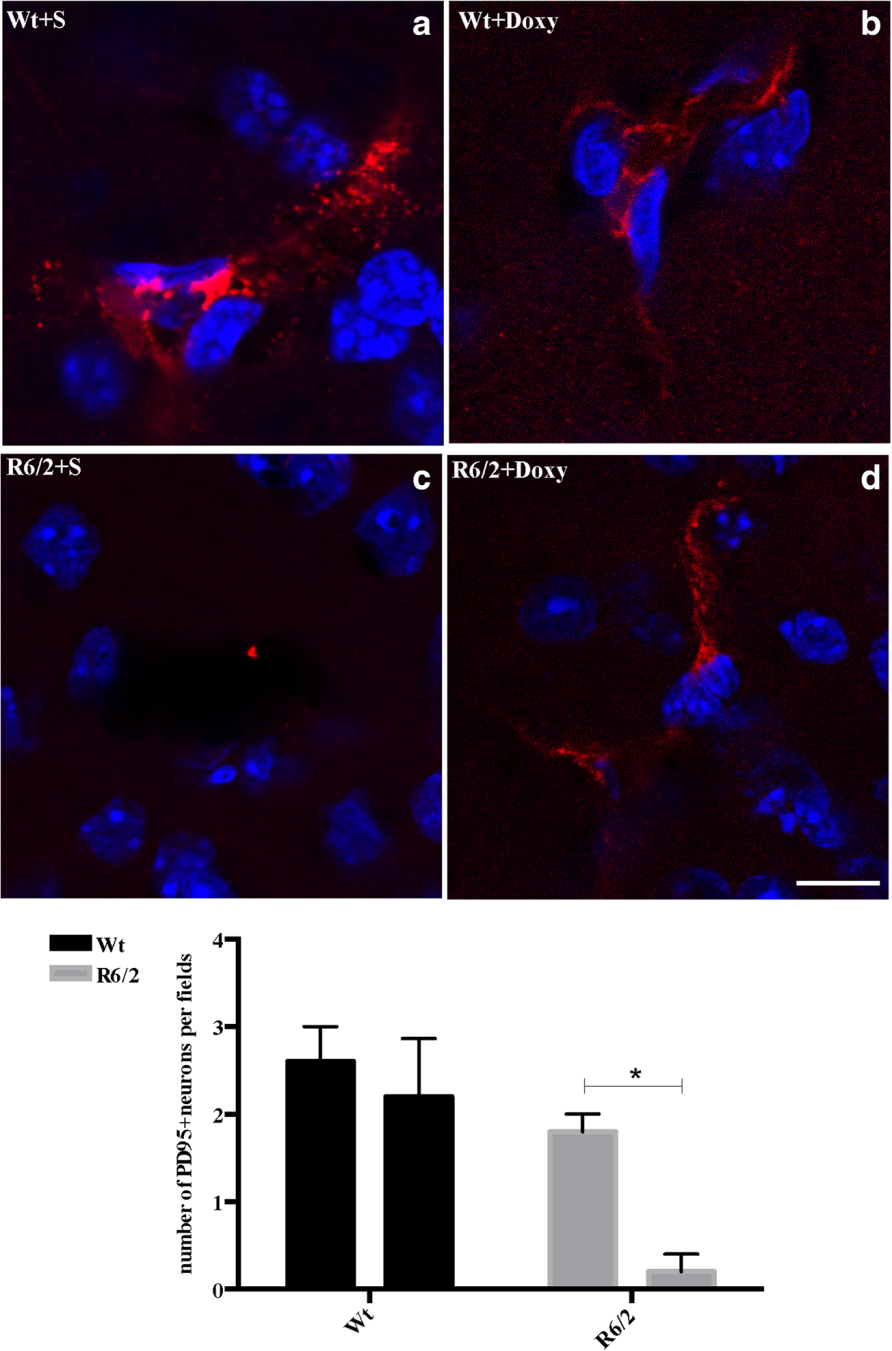
Doxycycline prevents post-synaptic modification in R6/2 mice. **a–d** High magnification (× 63) representative confocal images show PSD95 immunostaining in all experimental groups (*scale bar 10* μm) (*n* = 2 female and *n* = 2 male mice for each group; 3 brain sections for mice). **d** Analyzed data revealed that doxycycline was able to retain the expression of PSD95 in the R6/2 mice when compared to R6/2 treated with saline *F*_1,12_ = 2.774 *p* = 0.022. Histograms are mean ± SEM of PSD95 quantification

In order to examine whether doxycycline could protect synapses, we investigated the expression of the post-synaptic marker PSD-95, through immunofluorescence. As shown in Fig. 8, we found that doxycycline-treated mice significantly retain the expression of PSD95 compared to the saline-treated one indicating the ability of doxycycline to preserve synapses in R6/2 mice; (*n* = 4, mean ± SEM, *p* < 0.05).

##### Doxycycline Reduced Microglia Activation in R6/2 Mice

**Fig. 9:**
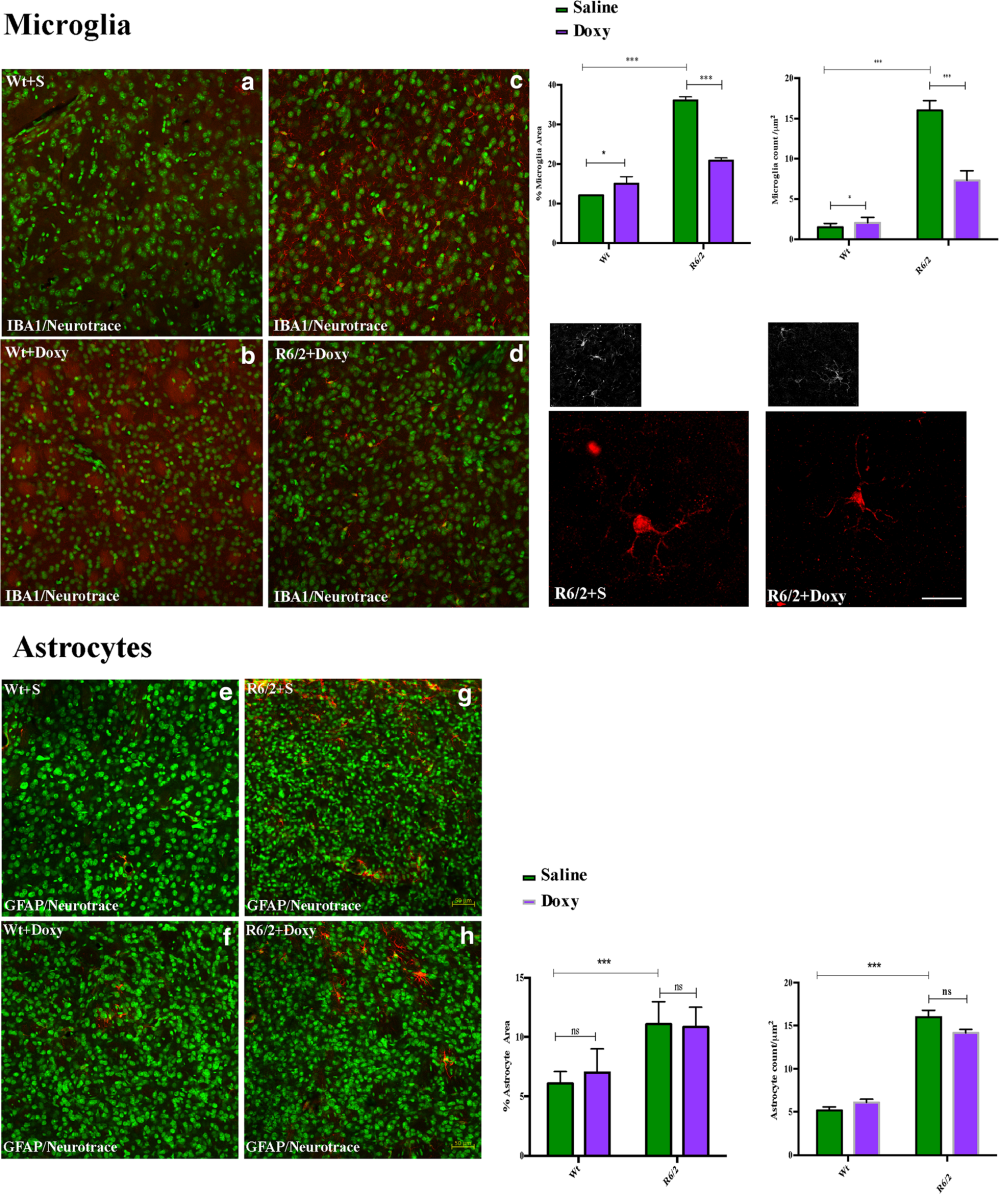
Doxycycline reduces microglial activation, but had no effect on astrogliosis. **a** Representative confocal images showing the distribution of microglia in the four experimental groups (*n* = 8 female and *n* = 5 male mice for each group; 5 brain sections for mice). **b** Significantly lower microglia count was recorded in the doxycycline-treated R6/2 mice with respect to the saline-treated one: *F*_1,24_ = 9747 *p* < 0.001. **c** Analyzed collected data revealed a significant reduction of microglia area in the R6/2 mice treated with doxycycline with respect to saline-treated R6/2 mice *F*_1,24_ = 239.1 *p* < 0.001. **d** Higher magnification representative confocal images showing the soma size of Iba-1 positive cells in R6/2 saline- (left panels) or doxycycline-treated mice (right panels). **e** Representative confocal images showing the distribution of astrocytes in the four experimental animal groups. Student *T* test analysis showed that GFAP positive cells (astrocytes) increased significantly in the saline-treated R6/2 mice compared to wild-type littermates: *F*_1,24_ 178.5 *p* < 0.001 and doxycycline had no significant effect on both number (**f**) and soma’s size (**g**-**h**) *F*_1,24_ = 0.5264, *F*_1,24_ = 0.2829 *p* = 0.5997 respectively

Iba-1 immunofluorescence was performed to address the different activation stages of microglia. Wild-type mice treated with saline or with doxycycline displayed a ramified or primed microglia which show a bigger cell body but with the similar ramification of ramified microglia. The presence of dystrophic microglia in the wild-type mice can be attributed to the aging/senescence process. Moreover, we can also expect that the chronic treatment with saline or doxycycline can be related to a partial state of inflammation (Suppl.1). The immunostaining for Iba-1 in the saline-treated R6/2 group revealed an intense microglial reaction, where microglial cells appeared numerous and displayed an amoeboid cell body in which are still present few ramified or unramified processes (Fig. 9c). Microglial reaction appeared markedly attenuated in doxycycline-treated R6/2 mice, with fewer reactive Iba-1 positive cells and a smaller circular cell body with a ramification pattern that suggest a resting phenotype (Fig. 9d). Moreover, we investigated, through GFAP immunohistochemistry, the astrocytic reaction in saline or doxycycline-treated R6/2 mice. Statistical analysis revealed that the higher degree of astrocytosis observed in R6/2 mice treated with saline compared to wild-type mice was not modified by doxycycline administration; (Fig. 9g, h).

## Discussion

The sum of our data show that administration of doxycycline is protective in the R6/2 mouse model of HD in terms of survival, motor performance, and neuroprotection. Of note, the positive effects induced by doxycycline were associated to a significant decrease in the extent of microglial activation.

R6/2 mice treated with doxycycline lived significantly longer and displayed healthier conditions compared to saline-treated mice. At a functional level, we show that doxycycline significantly delayed the onset and the severity of motor dysfunctions in R6/2 mice tested on rotarod and in the open field. This effect is compelling, when one considers that motor activity recovery is a vital therapeutic target in HD.

At a neuropathological level, we found that doxycycline significantly reduced the number of NIIs in R6/2 striatal neurons. The ability of doxycycline to drastically reduce aggregation of huntingtin exon 1 at a concentration of 30 μM was previously shown in organotypic slice culture [22]. However, we speculate that the protective effects in R6/2 HD mice might not reside exclusively in an effect on aggregation. Likely, the anti-inflammatory effect might be most relevant, as previously demonstrated in Alzheimer’s disease mouse models [16, 17]. This effect is confirmed in the R6/2 mouse model of HD as per the evidence that doxycycline drastically reduced the number and activation of microglial cells.

At a cellular level, we also proved the ability of the drug to positively modulate CREB activity and the expression of BDNF. The vulnerability of medium spiny neurons of the striatum to Huntington’s disease degeneration is postulated to be caused by a transcriptional dysregulation of cAMP and CREB signaling cascades.

Indeed, a downregulation of CREB-mediated transcription has been hypothesized to contribute to neuronal loss in HD [28–31]. In addition, a decreased transcription of CREB-regulated genes occurs in HD mouse models [29, 32, 33]. Therefore, preventing the decreased cAMP signaling and loss of CREB-regulated gene transcription represents a valid therapeutic strategy for HD [25].

Mutated huntingtin has been shown to interfere with some polyglutamine containing transcription factors. In particular, the detrimental interaction with CREB-binding protein (CPB) was described earlier [34]. Notably, in our study, doxycycline promoted cell survival and was associated with an upregulation of phosphorylated CREB. CREB induces transcription of about 4000 target genes, including genes regulating apoptosis [35, 36]. Indeed, a disruption of signaling cascades plays a key role in the pathology caused by mutant huntingtin in both transgenic mice and HD patients. One of the key downstream mediators in this regard is BDNF, a principal neurotrophic factor for both striatal and cortical neurons. Interestingly, among the target genes, BDNF is the most affected in HD [27].

A distinct involvement of BDNF was demonstrated in the pathophysiology of HD, where a loss of huntingtin-mediated BDNF gene transcription was described both in animal models and in patients [27]. Moreover, BDNF knockout mice display an earlier age of onset and more severe motor symptoms [37]. Conversely, BDNF administration proved to be beneficial in several disease models [[38, 39]) including HD ([40, 41]).

These data support the evidence that BDNF plays a role in the discrete degeneration of striatal projection neurons in HD. Lower serum levels of BDNF were described in HD patients compared to controls, and the severity of clinical signs negatively correlated with levels of BDNF [42].

In the present study, we found that R6/2 mice treated with doxycycline displayed a higher expression of BDNF. We interpret the higher CREB phosphorylation and BDNF expression observed in our study to be, at least in part, due to the activation by doxycycline of pro-survival mechanisms. Of note, while CREB phosphorylation promotes an increase in BDNF, we have previously shown that also BDNF administration, possibly by a positive feedback mechanism, results in an increased CREB phosphorylation [40].

It is conceivable that all these positive neuronal changes, some of which could be also attributed to the lower transgene expression, together with the decrease in microglial activation, mediated by doxycycline, participate to rescue neuronal activity [43]. Moreover, the potential effects of doxycycline on the gut microbiome could be taken into account when speculating on the beneficial action of the compound. Indeed, a gut dysbiosis occurs in Huntington’s disease ([44]); thus, an influence of antibiotics on the HD phenotype could be expected.

Another tetracycline, namely, minocycline, was previously demonstrated to be neuroprotective in several models of disease ([45]) and was also tested in HD models ([46, 47]), mainly for its anti-apoptotic properties, and secondarily for its anti-inflammatory activity. In the study by Chen and coauthors (2000), the administration of minocycline was associated with the delay of disease progression. In a subsequent study [22], as mentioned before, the inhibition of aggregation of mutated huntingtin by minocycline and doxycycline was not associated with beneficial effects in terms of behavioral abnormalities. The debate about effects of tetracyclines, however, has continued for several years, with some authors still affirming their neuroprotective effects ([47–49]).

Indeed, the study by Smith and coauthors (2003) measuring the effects of doxycycline in the R6/2 mice had led to disappointing results. However, the route of administration used in that study was different, since they used doxycycline orally instead of IP. The expected brain levels of doxycycline, according to the study by Lucchetti et al. [50], are of 0.22 μg/g [50]. This could possibly explain, at least in part, the discrepancy in the outcomes. In addition, the 2003 study lacked measurements of survival, which we found to be significantly elongated by the treatment, and a series of other primary outcome measures, that we found to be positively changed by doxycycline in our study. In fact, the neuronal size and number was significantly higher in R6/2 treated with doxycycline, and the number and size of NIIs were significantly decreased.

Further support to the neuroprotective action of doxycycline comes from the evidence that PSD-95 immunoreactivity in the striatum of the wild type and of the R6/2 mice treated with doxycycline were higher than R6/2 mice treated with saline.

PSD-95 is an important scaffolding protein in the post-synaptic density (PSD) of dendritic spines. Here, PSD-95 stabilizes glutamate receptors at the sites of synaptic transmission. Moreover, PSD-95 forms ternary protein complexes with D1 and NMDA receptors, and plays a role in limiting the reciprocal potentiation between both receptors from increasing excessively. Mice lacking PSD-95, resulting from genetic deletion of the GK domain of PSD-95, develop a progressive neurological syndrome that includes hypolocomotion, limb clasping, and from a histological point of view, major loss of spiny projection neurons. Therefore, a protective role for PSD-95 was suggested [51].

Taken together, our data strongly indicate that doxycycline can be considered as an attractive candidate compound for HD, benefitting from its long history of safe use in clinical settings. Therefore, it could speedily bypass many of the early safety trials and pass rapidly into routine clinical use.

## Electronic Supplementary Material

ESM 1 (PNG 18099 kb)

High Resolution Image (TIFF 3056 kb)

## References

[1] Biber K, Möller T, Boddeke E, Prinz M (2016). Central nervous system myeloid cells as drug targets: current status and translational challenges. Nat Rev Drug Discov.

[2] Kim EK, Choi E-J (2015). Compromised MAPK signaling in human diseases: an update. Arch Toxicol.

[3] Block ML, Hong J-S (2007). Chronic microglial activation and progressive dopaminergic neurotoxicity. Biochem Soc Trans.

[4] Pawate S, Shen Q, Fan F, Bhat NR (2004). Redox regulation of glial inflammatory response to lipopolysaccharide and interferongamma. J Neurosci Res.

[5] Dik B, Coskun D, Bahcivan E, Er A (2019). Doxycyclinecycline and meloxicam can treat neuroinflammation by increasing activity of antioxidant enzymes in rat brain. Pak J Pharm Sci.

[6] Santa-Cecilia FV, Socias B, Ouidja MO, Sepulveda-Diaz JE, Acuna L, Silva RL, Michel PP, Del-Bel E, Cunha TM, Raisman-Vozari R (2016). Doxycyclinecycline suppresses microglial activation by inhibiting the p38 MAPK and NF-kB signaling pathways. Neurotox Res.

[7] Forloni G, Colombo L, Girola L, Tagliavini F, Salmona M (2001). Anti-amyloidogenic activity of tetracyclines: studies in vitro. FEBS Lett.

[8] Tagliavini F, Forloni G, Colombo L, Rossi G, Girola L, Canciani B, Angeretti N, Giampaolo L, Peressini E, Awan T, De Gioia L, Ragg E, Bugiani O, Salmona M (2000). Tetracycline affects abnormal properties of synthetic PrP peptides and PrP(Sc) in vitro. J Mol Biol.

[9] De Luigi A, Colombo L, Diomede L, Capobianco R, Mangieri M, Miccolo C, Limido L, Forloni G, Tagliavini F, Salmona M (2008). The efficacy of tetracyclines and intracerebral prion infection. PLoS One.

[10] Forloni G, Iussich S, Awan T, Colombo L, Angeretti N, Girola L, Bertani I, Poli G, Caramelli M, Grazia Bruzzone M, Farina L, Limido L, Rossi G, Giaccone G, Ironside JW, Bugiani O, Salmona M, Tagliavini F (2002). Tetracyclines affect prion infectivity. Proc Natl Acad Sci U S A.

[11] Forloni G, Salmona M, Marcon G, Tagliavini F (2009). Tetracyclines and prion infectivity. Infect Disord Drug Targets.

[12] Stoilova T, Colombo L, Forloni G, Tagliavini F, Salmona M (2013). A new face for old antibiotics: tetracyclines in treatment of amyloidoses. J Med Chem.

[13] Brundula V, Rewcastle NB, Metz LM, Bernard CC, Yong VW (2002). Targeting leukocyte MMPs and transmigration: minocycline as a potential therapy for multiple sclerosis. Brain J Neurol.

[14] Krakauer T, Buckley M (2003). Doxycyclinecycline is anti-inflammatory and inhibits staphylococcal exotoxin-induced cytokines and chemokines. Antimicrob Agents Chemother.

[15] Wang Z, Xue Y, Jiao H, Liu Y, Wang P (2012). Doxycyclinecycline-mediated protective effect against focal cerebral ischemia-reperfusion injury through the modulation of tight junctions and PKCδ signaling in rats. J Mol Neurosci.

[16] Balducci C, Santamaria G, La Vitola P, Brandi E, Grandi F, Viscomi AR, Beeg M, Gobbi M, Salmona M, Ottonello S, Forloni G (2018). Doxycycline counteracts neuroinflammation restoring memory in Alzheimer’s disease mouse models. Neurobiol Aging.

[17] Balducci C, Santamaria G, La Vitola P, Brandi E, Grandi F, Viscomi AR, Beeg M, Gobbi M, Salmona M, Ottonello S, Forloni G (2018). Doxycyclinecycline counteracts neuroinflammation restoring memory in Alzheimer’s disease mouse models. Neurobiol Aging.

[18] Assar H, Topakian R, Weis S, Rahimi J, Trenkler J, Hoftberger R, Aboulenein Djamshidian F, Strobel T, Budka H, Yull H, Head MW, Ironside JW, Kovacs GG (2015). A case of variably protease-sensitive prionopathy treated with doxycyclinecyclin. J Neurol Neurosurg Psychiatry.

[19] Pocchiari M, Ladogana A (2015). Rethinking of doxycyclinecycline therapy in Creutzfeldt-Jakob disease. J Neurol Neurosurg Psychiatry.

[20] Varges D, Manthey H, Heinemann U, Ponto C, Schmitz M, Schulz-Schaeffer WJ, Krasnianski A, Breithaupt M, Fincke F, Kramer K, Friede T, Zerr I (2017). Doxycyclinecycline in early CJD: a double-blinded randomised phase II and observational study. J Neurol Neurosurg Psychiatry.

[21] Minagar A, Alexander JS, Schwendimann RN, Kelley RE, Gonzalez-Toledo E, Jimenez JJ, Mauro L, Jy W, Smith SJ (2008). Combination therapy with interferon beta-1a and doxycyclinecycline in multiple sclerosis: an open-label trial. Arch Neurol.

[22] Smith DL, Woodman B, Mahal A, Sathasivam K, Ghazi-Noori S, Lowden PA, Bates GP, Hockly E (2003). Minocycline and doxycyclinecycline are not beneficial in a model of Huntington’s disease. Ann Neurol.

[23] Hersch SM, Ferrante RJ (2004). Translating therapies for Huntington’s disease from genetic animal models to clinical trials. NeuroRx.

[24] DeMarch Z, Giampà C, Patassini S, Martorana A, Bernardi G, Fusco FR (2007). Beneficial effects of rolipram in a quinolinic acid model of striatal excitotoxicity. Neurobiol Dis.

[25] Giampà C, DeMarch Z, D'Angelo V, Morello M, Martorana A, Sancesario G, Bernardi G, Fusco FR (2006). Striatal modulation of cAMP-response-element-binding protein (CREB) after excitotoxic lesions: implications with neuronal vulnerability in Huntington's disease. Eur J Neurosci.

[26] Paldino E, Cardinale A, D'Angelo V, Sauve I, Giampà C, Fusco FR (2017). Selective sparing of striatal interneurons after poly (ADP-ribose) polymerase 1 inhibition in the R6/2 mouse model of Huntington’s disease. Front Neuroanat.

[27] Zuccato C, Ciammola A, Rigamonti D, Leavitt BR, Goffredo D, Conti L, MacDonald ME, Friedlander RM, Silani V, Hayden MR, Timmusk T, Sipione S, Cattaneo E (2001). Loss of huntingtin-mediated BDNF gene transcription in Huntington’s disease. Science.

[28] Jiang H, Nucifora FC, Ross CA, DeFranco DB (2003). Cell death triggered by polyglutamine-expanded huntingtin in a neuronal cell line is associated with degradation of CREB-binding protein. Hum Mol Genet.

[29] Nucifora FC, Sasaki M, Peters MF, Huang H, Cooper JK (2001). Interference by huntingtin and atrophin-1 with cbp-mediated transcription leading to cellular toxicity. Science..

[30] Steffan JS, Kazantsev A, Spasic-Boskovic O, Greenwald M, Zhu YZ (2000). The Huntington’s disease protein interacts with p53 and CREB-binding protein and represses transcription. Proc Natl Acad Sci U S A.

[31] Steffan JS, Bodai L, Pallos J, Poelman M, McCampbell A (2001). Histone deacetylase inhibitors arrest polyglutamine-dependent neurodegeneration in Drosophila. Nature..

[32] Luthi-Carter R, Strand A, Peters NL, Solano SM, Hollingsworth ZR (2000). Decreased expression of striatal signaling genes in a mouse model of Huntington’s disease. Hum Mol Genet.

[33] Wyttenbach A, Swartz J, Kita H, Thykjaer T, Carmichael J (2001). Polyglutamine expansions cause decreased CRE-mediated transcription and early gene expression changes prior to cell death in an inducible cell model of Huntington’s disease. Hum Mol Genet.

[34] Giralt A, Puigdellívol M, Carretón O, Paoletti P, Valero J, Parra-Damas A, Saura CA, Alberch J, Ginés S (2012). Long-term memory deficits in Huntington’s disease are associated with reduced CBP histone acetylase activity. Hum Mol Genet.

[35] Siu Y-T, Jin D-Y (2007). CREB—a real culprit in oncogenesis. FEBS J.

[36] Zhang X, Odom DT, Koo S-H, Conkright MD, Canettieri G, Best J, Chen H, Jenner R, Herbolsheimer E, Jacobsen E, Kadam S, Ecker JR, Emerson B, Hogenesch JB, Unterman T, Young RA, Montminy M (2005). Genome-wide analysis of cAMP-response element binding protein occupancy, phosphorylation, and target gene activation in human tissues. Proc Natl Acad Sci U S A.

[37] Canals JM, Pineda JR, Torres-Peraza JF, Bosch M, Martín-Ibañez R (2004). Brain-derived neurotrophic factor regulates the onset and severity of motor dysfunction associated with enkephalinergic neuronal degeneration in Huntington’s disease. J Neurosci.

[38] Nakao N, Brundin P, Funa K, Lindvall O, Odin P (1995). Trophic and protective actions of brain-derived neurotrophic factor on striatal DARPP-32-containing neurons in vitro. Dev Brain Res.

[39] Petersen A, Larsen KE, Behr GG, Romero N, Przedborski S (2001). Brain-derived neurotrophic factor inhibits apoptosis and dopamine-induced free radical production in striatal neurons but does not prevent cell death. Brain Res Bull.

[40] Giampà C, Montagna E, Dato C, Melone MAB, Bernardi G, Fusco FR (2013). Systemic delivery of recombinant brain derived neurotrophic factor (BDNF) in the R6/2 mouse model of Huntington’s disease. PLoS One.

[41] Paldino E, Giampà C, Montagna E, Angeloni C, Fusco FR (2019) Modulation of Phospho-CREB by Systemically Administered Recombinant BDNF in the Hippocampus of the R6/2 Mouse Model of Huntington's Disease. Neurosci J 8363274. 10.1155/2019/8363274 10.1155/2019/8363274 PMC6381568 30881980

[42] Ciammola A, Sassone J, Cannella M, Calza S, Poletti B (2007). Low brain-derived neurotrophic factor (BDNF) levels in serum of Huntington’s disease patients. Am J Med Genet B Neuropsychiatr Genet.

[43] Cardinale A, Paldino E, Giampà C, Bernardi G, Fusco FR (2015). PARP-1 inhibition is neuroprotective in the R6/2 mouse model of Huntington’s disease. PLoS One.

[44] Kong G, Cao KL, Judd LM, Li S, Renoir T, Hannan AJ (2018). Microbiome profiling reveals gut dysbiosis in a transgenic mouse model of Huntington’s disease. Neurobiol Dis.

[45] Zhang SC, Goetz BD, Duncan ID (2003) Suppression of activated microglia promotes survival and function of transplanted oligodendroglial progenitors. Glia 41(2):191–8 10.1002/glia.10172 12509809

[46] Chen M, Ona VO, Li M, Ferrante RJ, Fink KB, Zhu S, Bian J, Guo L, Farrell LA, Hersch SM, Hobbs W, Vonsattel JP, Cha JH, Friedlander RM (2000) Minocycline inhibits caspase-1 and caspase-3 expression and delays mortality in a transgenic mouse model of Huntington disease. Nat Med 6(7):797–801 10.1038/77528 10888929

[47] Stack EC, Smith KM, Ryu H, Cormier K, Chen M, Hagerty SW, Del Signore SJ, Cudkowicz ME, Friedlander RM, Ferrante RJ (2006) Combination therapy using minocycline and coenzyme Q10 in R6/2 transgenic Huntington's disease mice. Biochim Biophys Acta 1762(3):373–80 10.1016/j.bbadis.2005.11.002 16364609

[48] Sancho M1, Herrera AE, Gortat A, Carbajo RJ, Pineda-Lucena A, Orzáez M, Pérez-Payá E (2011) Minocycline inhibits cell death and decreases mutant Huntingtin aggregation by targeting Apaf-1. Hum Mol Genet 20(18):3545–53. 10.1093/hmg/ddr271 10.1093/hmg/ddr271 21659333

[49] Kumar A, Chaudhary T, Mishra J (2013) Minocycline modulates neuroprotective effect of hesperidin against quinolinic acid induced Huntington's disease like symptoms in rats: behavioral, biochemical, cellular and histological evidences. Eur J Pharmacol 720(1–3):16–28. 10.1016/j.ejphar.2013.10.057 10.1016/j.ejphar.2013.10.057 24211676

[50] Lucchetti J, Fracasso C, Balducci C, Passoni A, Forloni G, Salmona M, Gobbi M (2019). Plasma and brain concentrations of doxycycline after single and repeated doses in wild-type and APP23 mice. J Pharmacol Exp Ther.

[51] Zhang J, Saur T, Duke AN, Grant SGN, Platt DM, Rowlett JK, Isacson O, Yao W-D (2014). Motor impairments, striatal degeneration, and altered dopamine-glutamate interplay in mice lacking PSD-95. J Neurogenet.

