# Supplementary figures and images for: Neuroprotective Effects of Doxycycline in the R6/2 Mouse Model of Huntington’s Disease
Source: Mol Neurobiol. 2019 Dec 26;57(4):1889–903. doi: 10.1007/s12035-019-01847-8 (PMC7118056; doi:10.1007/s12035-019-01847-8)

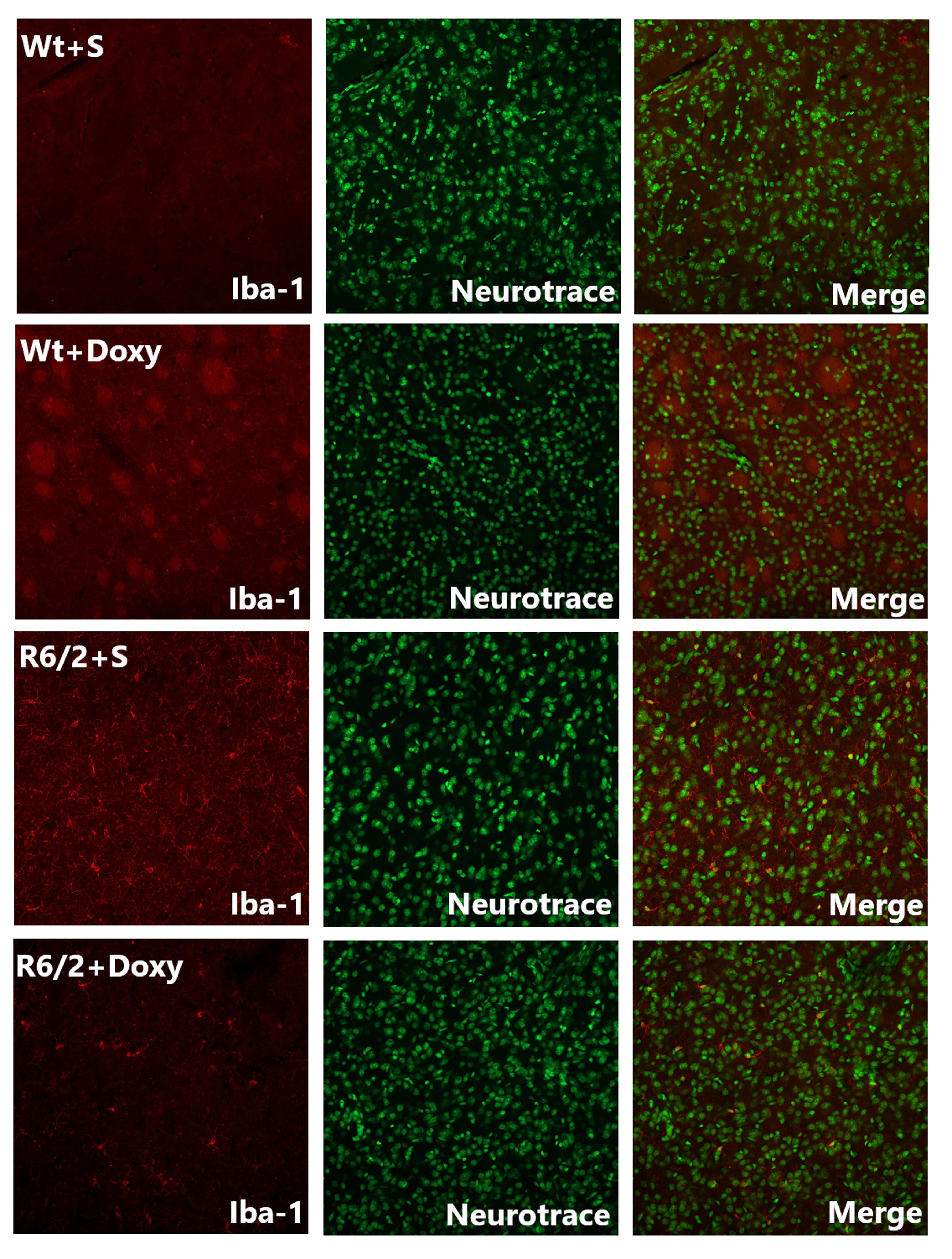

Supplement: Supplementary file 1 — (PNG 18099 kb) [file 12035_2019_1847_Fig10_ESM.png]

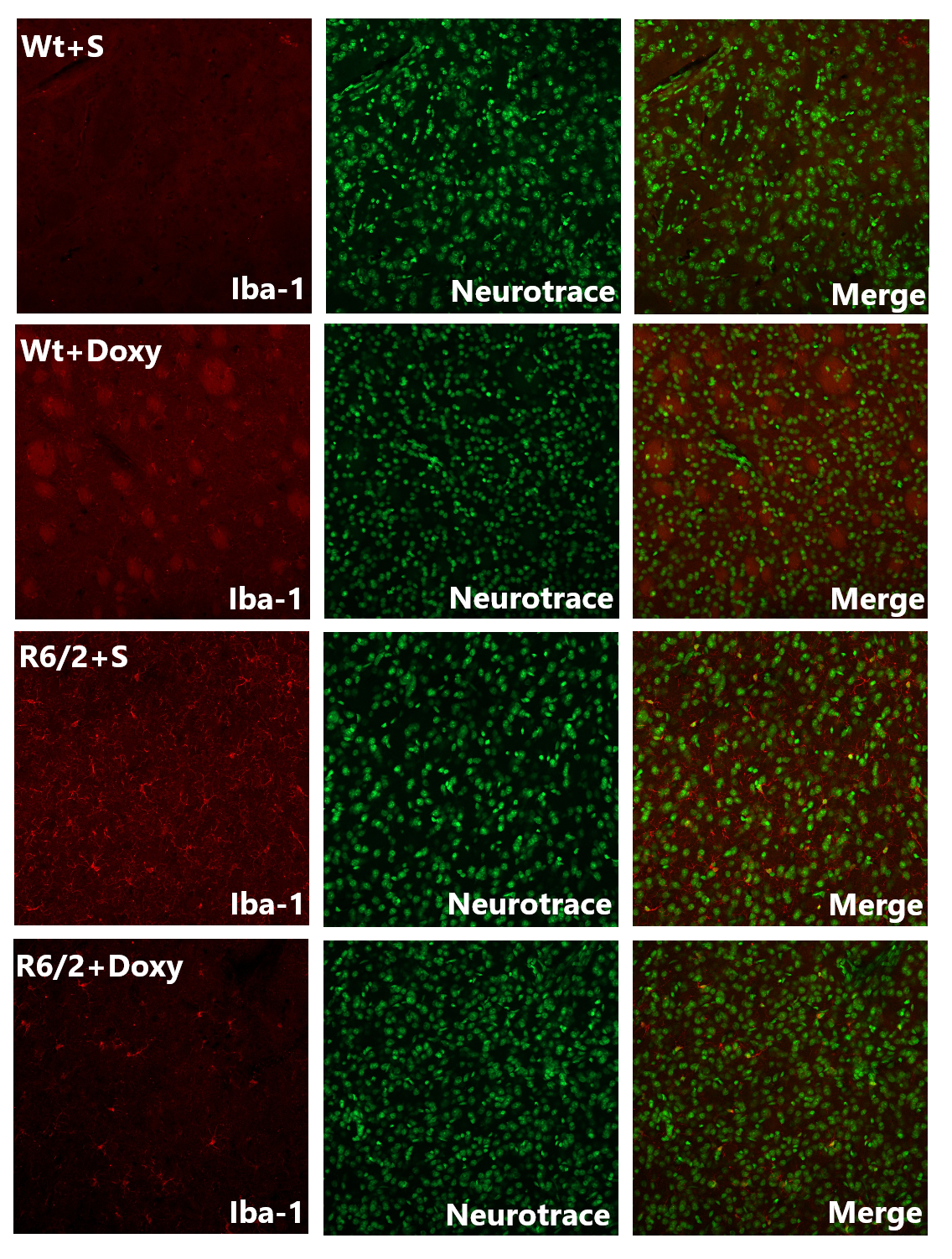

Supplement: Supplementary file 2 — High Resolution Image (TIFF 3056 kb) [file 12035_2019_1847_MOESM1_ESM.tiff]
